# The health profile of newly-arrived refugee women and girls and the role of region of origin: using a population-based dataset in California between 2013 and 2017

**DOI:** 10.1186/s12939-019-1066-3

**Published:** 2019-10-16

**Authors:** May Sudhinaraset, Nuny Cabanting, Marisa Ramos

**Affiliations:** 1 Jonathan and Karin Fielding School of Public Health, University of California, Los Angeles, 650 Charles E Young Dr. S, Los Angeles, CA 90095 USA; 2 Office of Refugee Health, Center for Infectious Disease, California Department of Public Health, 1616 Capitol Ave, Sacramento, CA 95899 USA

**Keywords:** Refugee, Health status, Region of origin, Women, Girls, Female, Trauma

## Abstract

**Background:**

There has been an increasing number of refugee women globally; yet, there is little recent data describing the health profile of refugee women by region of origin in the United States. It is important to monitor the health status of women by region of origin to provide needed targeted interventions.

**Methods:**

We analyzed the Refugee Health Electronic Information System (RHEIS), a population-based dataset that included 14,060 female refugees who entered California between October 3, 2013 and February 15, 2017. We assessed differences in health status by region of origin.

**Results:**

Almost one out of three women experienced a traumatic event. Women from Africa and Latin America and the Caribbean experienced higher levels of trauma compared to other regions, including sexual assault, physical, and weapon assault. More than half of women and girls (56.6%) reported experiences of persecution, with Southeast Asians reporting the highest levels. Among women of reproductive age, 7.0% of women were currently pregnant at the time of arrival to the US, 19.0% ever had a spontaneous abortion, and 8.6% reported ever having an abortion. One in three women from Africa reported female genital cutting. Moreover, 80.0% of women reported needing language assistance at the time of their health assessment.

**Conclusions:**

Refugee women and girls experience high levels of trauma and persecution, suggesting the need for trauma-informed care. Those working with refugee women, such as resettlement agencies and health providers, should be equipped with information about antenatal care, nutrition, and pregnancy to newly arrived women. Lastly, differences in health status by region of origin indicate a need for tailored interventions and linguistically appropriate health information.

## Background

According to the United Nations High Commissioner for Refugees (UNHCR), over 65 million people were forced to flee their homes in 2015-- an unprecedented number that is reflective of broader political and social turmoil occurring globally [1]. This includes over 21 million individuals with refugee status, with 54% of refugees from three countries: Syria, Afghanistan, and Somalia [2]. In the United States (US), the total number of refugees admitted fluctuates with ongoing political priorities and global events, with one of the highest numbers reported recently in fiscal year 2016 when 84,995 refugees were admitted [3]. While this number has declined since 2017, refugees continue to be an important population in the US given that thousands will continue to resettle each year coming from diverse regions of the globe. Moreover, the screening and vetting process is intense, lasting an average of two years [4]. First, the UNHCR refers cases to be considered for resettlement to countries and provides background information. The US then screens and decides whether refugees are admitted based on a thorough vetting process that includes multiple government agencies, security databases, background checks, and in-person interviews [4]. Historically, the United States has admitted refugees from all regions of the world, including Asia, Africa, Europe, Latin America, and the Middle East. The numbers of refugees from particular countries vary greatly depending on current political and social events. For example, in the early 1990s, refugees coming from former Soviet countries increased in response to the collapse of the Soviet Union; meanwhile, between 2006 and 2016, the highest numbers of refugees came from Myanmar, Iraq, and Bhutan due to ongoing wars and political and religious persecution [3]. California, in particular, has historically received one of the largest number of refugees in the country, with approximately 5100 refugees (9.3% of all new arrivals) in 2017 [5]. Newly arriving refugees in California represent an ethnically diverse group coming from all regions of the world, representing 85 countries and speaking more than 80 different languages [6]. The diversity of the refugee population contributes to California’s cultural and social identity; yet it also provides unique challenges for service providers and agencies working with refugee groups.

Moreover, women and children are particularly vulnerable as they are disproportionately exposed to conflict-related trauma in their country of origin and during migration [7]. Similar to men, newly arrived refugee women experience challenges related to: lack of knowledge about resources or protections available in the receiving country, language and cultural barriers, and unstable economic circumstances [8]. However, young women and girls experience increased vulnerability due to other factors including increased risk of sexual violence and trauma during the migration process [9], sexual trafficking, and increased needs related to obstetric and reproductive health care (i.e. pregnancies, miscarriages, abortion care, access to contraception, etc.) [9–12]. However, to our knowledge, there are no published results on the health status of refugee women and children.

This study makes use of a novel dataset to provide much needed information on refugee populations. The Refugee Act of 1980 passed by Congress mandated that all refugees entering the United States receive standardized medical care and earmarked federal funding for health services at the local and state levels. Consequently, California’s Office of Refugee Health developed the Refugee Health Assessment Program (RHAP), a multi-faceted program that aims to provide refugees with culturally competent medical services. In 2013, the RHAP standardized its data monitoring systems by implementing an electronic database –the Refugee Health Information System (RHEIS). This manuscript leverages this novel dataset in order to help guide local program planning and policy decisions.

Resettlement and government agencies engage intensively with refugees at the point of and within one year of arrival, offering support services such as cash assistance, medical assistance, housing, and employment assistance. Given the significant heterogeneity in the refugee population in the US today, it is important to examine the health concerns among refugees from different regions of the world. Most research on refugees has been small-scale in nature or focused on specific refugee populations [13–15]. The social, political, and historical contexts unique to countries and regions of the world create diverging flows of refugee populations certain to impact health outcomes differently.

The objective of this manuscript is to present a health profile of newly arrived refugee women and girls using data from California between 2013 and 2017. Because this represents one of the first population-based data of refugee women and children in California, our specific objectives are to provide baseline information on demographic indicators, health status and health behaviors, and trauma and persecution experiences of refugee women and children entering California in their first 90 days of arrival.

## Methods

### Data collection

Post-arrival health assessment data were collected pursuant to the Refugee Health Assessment Program (RHAP). Health assessments are conducted over the course of two visits in the first 90 days following a refugee’s arrival to one of the eleven refugee county clinics in California. The first visit involves a general medical evaluation that includes any current symptoms or concerns; past medical and obstetric history; a traumatic events assessment (only asked of individuals 16 and older); and a social history including lifestyle assessment asked of individuals 15 years and older (i.e. sexual practices, tobacco, alcohol, and drug use). Refugees receive a basic physical assessment at this visit, including vital signs, immunizations, and laboratory blood draws to screen for infectious diseases, micronutrient deficiencies, and chronic disease indicators and/or risk factors. The second visit includes more specific medical concerns, reviewing lab results, and a general physical exam.

All information collected over the course of these two visits is documented on the California Refugee Health Assessment (CRHA) form and entered into the Refugee Health Electronic Information System (RHEIS), an electronic database. A nurse practitioner or medical doctor fills out the health assessment form by paper and then is transferred to the RHEIS by an office staff. All clinics were trained on recording health indicators of interest and data entry into the RHEIS database.

### Study population, data and measures

In total, 14,060 women and girls who entered California between October 3, 2013 and February 15, 2017 initiated the health assessment. All underwent a full or partial medical assessment at a refugee clinic in one of 11 counties in California, representing all potential sites for health assessments in California. According to Centers for Disease Control (CDC) guidelines, all refugees are recommended to take a post-arrival health assessment. This reported data represents approximately 95% of all refugees entering California during this time, and is the most comprehensive datasets on refugees in the state [16]. For the analytic sample, we include only women and girls who completed the health assessment (*n* = 12,277). Additionally, because we were interested in the role of region of origin, we dropped individuals who had missing data on this indicator. We also dropped one person from Oceania region. In total, the analytic sample was 12,270.

Because this data represents monitoring and surveillance activities, there is a high degree of missing values. Table 1 includes a list of measures, reports missing data, and indicates which questions are asked of specific age groups. Demographic data were collected from all women and included entry status (refugee, asylum seekers, Special Immigrant Visas (SIVs), parolees, and victims of trafficking (VOT)). In this paper, we refer to individuals who come as refugee, asylum seekers, SIV, parolees, and VOT as “refugees.” Refugees are individuals who are unable or unwilling to return to their country of nationality due to persecution or a well-founded fear of persecution; asylees are those already in the US or at a port of entry who unable or unwilling to return to their country of nationality due to persecution or a well-founded fear of persecution; parolees are those who are allowed into the US for humanitarian reasons or that the entry of the individual is believed to be a significant public benefit; SIVs are individuals who worked for the United States in Iraq or Afghanistan as interpreters or providing other services; VOTs are individuals subjected to force, fraud or coercion for the purpose of sexual exploitation or forced labor [16]. Other demographic data included arrival year; age categories; whether an interpreter was needed to conduct the health assessment; parity (number of births); and whether an individual lived in another country prior to the US. Women were asked to indicate their countries of birth. Countries were then categorized into regions classified by the US State Department’s Bureau of Population, Refugees, and Migration (PRM) [17]. Regions of origin include Africa, Europe/Central Asia, Southeast Asia, South Asia, and Latin America and the Caribbean.

**Table 1:** Missing values for all variables

Variable	Missing No. (%)	Total
Age categories	3 (0)	12,270
Entry status	0 (0)	12,270
Education categories	1185 (9.7)	12,270
Interpreter needed	0 (0)	12,270
Lived in another country prior to US	0 (0)	12,270
Elevated glucose	2562 (20.9)	12,270
Ever smoke^a^	65 (0.7)	8751
Current smoker^a^	8014 (91.6)	8751
One alcohol drink^a^	65 (0.7)	8751
Pregnant result^a^	2769 (31.6)	8751
Experience of spontaneous abortion^a^	2162 (24.7)	8751
Abortion in lifetime^a^	2577 (29.4)	8751
Gravida: number of pregnancies^a^	582 (6.7)	8751
Parity: number of births woman has had^a^	990 (11.3)	8751
Hep B surface antigen^a^	154 (1.8)	8751
Hep B core antibody^a^	2359 (27)	8751
Hep B surface antibody^a^	2415 (27.6)	8751
Hep C^a^	1474 (16.8)	8751
HIV^a^	242 (2.8)	8751
Female genital cutting^a^	925 (10.6)	8751
Syphilis VDRL or RPR^a^	360 (4.1)	8751
Chlamydia^c^	522 (22.6)	2313
Any trauma experience^b^	152 (1.8)	8563
Persecution^b^	186 (2.2)	8563

Indicates specific age groups for which questions were asked: ^a^Age ≥ 15, ^b^ Age ≥ 16, ^c^Age ≥ 16 to Age ≤ 25

Women were asked about their histories of trauma throughout their life. Measures of traumatic events included: 1) physical assault (e.g. being attacked, hit, slapped, beaten up); 2) weapon assault (e.g. being shot, stabbed, threated with a knife, gun, bomb, land mine); 3) sexual assault (e.g. rape, attempted rape, made to perform any type of sexual act through force or threat of harm); 4) captivity (e.g. being kidnapped, abducted, held hostage, prisoner of war, forced labor); 5) sudden violent death of a family member (e.g. homicide, suicide); 6) serious injury, harm, or death you caused to someone else; 7) sudden move or loss of home and possessions.

Reproductive health indicators include current pregnancy status, lifetime experience of miscarriages, abortion, and female genital cutting. Current pregnancy status was confirmed through a urinary pregnancy test (classified as yes/no). Miscarriages classified the number of spontaneous abortions that the woman has had. At the health assessment, women were also asked about the number of abortions she has had. Miscarriages and abortions were categorized as binary measures and classified as ever experience spontaneous abortion or abortion. Female genital cutting was either self-reported or in a subset of instances depending on the health center, physicians also performed a visual check if the patient consented [we are not able to tell from the data how many visual checks were performed].

### Analysis

Univariate analyses were conducted on each variable to assess missing data and explore distributions of responses. Second, bivariate analyses were conducted for demographic characteristics and health outcomes by country of origin. We reported chi-2 statistics to determine statistical differences across regions of origin. All analyses were conducted using Stata 12MP.

### Ethical considerations

Data from these analyses are exempt from ethical review because they are part of California Department of Public Health’s routine data collection and monitoring activities as determined and reviewed by UCLA’s Institutional Review Board.

## Results

### Demographic characteristics

Out of 12,279 female women and girls, 13.4% were between the ages of 0–5 years, with more girls arriving from Europe/Central Asia and South Asia (16.7 and 14.0%, respectively) compared to other regions. The majority of individuals enter as refugees (60.3%), followed by 24.3% as Special Immigrant Visas (SIV), 10% of asylees, 1.5% as parolees, and 0.7% came as victims of trafficking (VOT) (see Table 2). The overwhelming majority of female refugees are from South Asia (73.9%), followed by women from Southeast Asia (7.8%), Africa (7.4%), Latin America and the Caribbean (LAC) (4.4%), and Europe/Central Asia (6.4%). Moreover, 12.9% reported no education, 24.1% 1–7 years of education, 17.3% 8–11 years, 29.8% 12–14 years, and 15.9% 15+ years of education. There were high levels of interpreters needed at the time of the health assessment (85.0%). Female refugees from Europe/Central Asia were most likely to need interpreter services (92.9%). Finally, 79.9% of women overall reported living in a transit country before arriving in the US.

**Table 2:** Demographics by Region of Birth

	Region of birth						
	Africa (Col %)	Southeast Asia (Col %)	Europe/Central Asia (Col %)	Latin America and the Caribbean (Col %)	South Asia (Col %)	Total (Col %)	*p*-value
Total	894 (100)	863 (100)	906 (100)	408 (100)	9199 (100)	12,270 (100)	
Age categories							0.000
0–5 years	110 (12.3)	85 (9.8)	151 (16.7)	13 (3.2)	1286 (14)	1645 (13.4)	
6–14 years	243 (27.2)	144 (16.7)	152 (16.8)	44 (10.8)	1291 (14)	1874 (15.3)	
15–24 years	183 (20.5)	161 (18.7)	138 (15.2)	121 (29.7)	1609 (17.5)	2212 (18)	
25–30 years	91 (10.2)	89 (10.3)	92 (10.2)	61 (15)	1356 (14.7)	1689 (13.8)	
31–45 years	178 (19.9)	240 (27.8)	175 (19.3)	107 (26.2)	1797 (19.5)	2497 (20.4)	
46–64 years	73 (8.2)	131 (15.2)	133 (14.7)	53 (13)	1356 (14.7)	1746 (14.2)	
65+ years	16 (1.8)	13 (1.5)	65 (7.2)	9 (2.2)	501 (5.4)	604 (4.9)	
Entry status							0.000
Asylee	183 (20.5)	538 (62.3)	75 (8.3)	137 (33.6)	332 (3.6)	1265 (10.3)	
Special Immigrant Visa (SIV)	0 (0)	24 (2.8)	18 (2)	6 (1.5)	2937 (31.9)	2985 (24.3)	
Parolee	0 (0)	0 (0)	0 (0)	178 (43.6)	0 (0)	178 (1.5)	
Refugee	708 (79.2)	247 (28.6)	811 (89.5)	67 (16.4)	5922 (64.4)	7755 (63.2)	
Victim of trafficking	3 (0.3)	54 (6.3)	2 (0.2)	20 (4.9)	8 (0.1)	87 (0.7)	
Education categories							0.000
None	202 (25.4)	86 (10.4)	74 (9.2)	8 (2.1)	1055 (12.8)	1425 (12.9)	
1–7 years	308 (38.7)	195 (23.6)	139 (17.2)	73 (18.8)	1960 (23.7)	2675 (24.1)	
8–11 years	114 (14.3)	193 (23.4)	235 (29.2)	81 (20.8)	1295 (15.7)	1918 (17.3)	
12–14 years	95 (11.9)	198 (24)	215 (26.7)	137 (35.2)	2659 (32.2)	3304 (29.8)	
15+ years	77 (9.7)	153 (18.5)	143 (17.7)	90 (23.1)	1300 (15.7)	1763 (15.9)	
Interpreter needed							0.000
No	301 (33.7)	268 (31.1)	64 (7.1)	121 (29.7)	1088 (11.8)	1842 (15)	
Yes	593 (66.3)	595 (68.9)	842 (92.9)	287 (70.3)	8111 (88.2)	10,428 (85)	
Lived in another country prior to US							0.000
No	153 (17.1)	427 (49.5)	206 (22.7)	191 (46.8)	1492 (16.2)	2469 (20.1)	
Yes	741 (82.9)	436 (50.5)	700 (77.3)	217 (53.2)	7707 (83.8)	9801 (79.9)	

### Experiences of trauma and persecution by region of birth

Importantly, almost one out of three women (27.7%) reported experiencing a traumatic event (Table 3). When looking across region, women from Africa and Latin America and the Caribbean have higher levels of trauma compared to other regions (44.5 and 41.2%, respectively). Women from Africa reported high levels of trauma related to sudden move/loss of home (21.2%), physical assault (17.0%), captivity (11.7%), sexual assault (10.5%), and weapon assault (17.4%). Women in Latin America and the Caribbean reported high levels of trauma related to assault including 28.4% of women reporting physical assault, 23.7% reporting weapon assault, and 17.0% reporting sexual assault.

**Table 3:** Experiences of trauma and persecution by Region of Birth

	Region of Birth						
	Africa (Col %)	Southeast Asia (Col %)	Europe/Central Asia (Col %)	Latin America and the Caribbean (Col %)	South Asia (Col %)	Total (Col %)	*p*-value
Total	523 (100)	611 (100)	593 (100)	342 (100)	6494 (100)	8563 (100)	
Any trauma experience							0.000
No trauma	287 (55.5)	490 (81)	496 (84.2)	197 (58.8)	4614 (72.5)	6084 (72.3)	
Any trauma experience	230 (44.5)	115 (19)	93 (15.8)	138 (41.2)	1751 (27.5)	2327 (27.7)	
*Specific type of trauma*							
Captivity							0.000
No	462 (88.3)	562 (92)	567 (95.6)	304 (88.9)	6052 (93.2)	7947 (92.8)	
Yes	61 (11.7)	49 (8)	26 (4.4)	38 (11.1)	442 (6.8)	616 (7.2)	
Harm or killed others							0.002
No	509 (97.3)	597 (97.7)	581 (98)	320 (93.6)	6255 (96.3)	8262 (96.5)	
Yes	14 (2.7)	14 (2.3)	12 (2.0)	22 (6.4)	239 (3.7)	301 (3.5)	
Death of a family member							0.000
No	449 (85.9)	588 (96.2)	562 (94.8)	296 (86.5)	5854 (90.1)	7749 (90.5)	
Yes	74 (14.1)	23 (3.8)	31 (5.2)	46 (13.5)	640 (9.9)	814 (9.5)	
Sudden move/loss of home							0.000
No	412 (78.8)	572 (93.6)	563 (94.9)	288 (84.2)	5432 (83.6)	7267 (84.9)	
Yes	111 (21.2)	39 (6.4)	30 (5.1)	54 (15.8)	1062 (16.4)	1296 (15.1)	
Physical assault							0.000
No	434 (83.0)	553 (90.5)	539 (90.9)	245 (71.6)	5929 (91.3)	7700 (89.9)	
Yes	89 (17.0)	58 (9.5)	54 (9.1)	97 (28.4)	565 (8.7)	863 (10.1)	
Sexual assault							0.000
No	468 (89.5)	592 (96.9)	581 (98)	284 (83)	6384 (98.3)	8309 (97)	
Yes	55 (10.5)	19 (3.1)	12 (2.0)	58 (17.0)	110 (1.7)	254 (3)	
Weapon assault							0.000
No	432 (82.6)	583 (95.4)	565 (95.3)	261 (76.3)	6078 (93.6)	7919 (92.5)	
Yes	91 (17.4)	28 (4.6)	28 (4.7)	81 (23.7)	416 (6.4)	644 (7.5)	
Persecution							0.000
No	164 (32.1)	117 (19.5)	473 (80.7)	98 (29.6)	2783 (43.8)	3635 (43.4)	
Yes	347 (67.9)	482 (80.5)	113 (19.3)	233 (70.4)	3567 (56.2)	4742 (56.6)	

Age ≥ 16 for all observations

Moreover, more than half of women and girls reported experiencing persecution in their countries (56.6%). Southeast Asian refugees were most likely to report persecution (80.5%), followed by refugees from Latin America and the Caribbean, Africa, South Asia, and Europe/Central Asia.

### Reproductive health indicators and infectious diseases by region of birth

There were 8751 women aged 15 years and older who were asked questions pertaining to reproductive health (Table 4). The mean number of births women had was 3.3, with women from Southeast Asia reporting the highest mean number of children (4.3) and Africa reporting the lowest (2.5). Women from Africa were most likely to report having no children (47.0%) compared to those from Europe/Central Asia (20.2). In total, 418 women reported being currently pregnant (7.0%), 1252 (19.0%) reported ever having a spontaneous abortion, and 531 (8.6%) reported ever having an abortion. Surprisingly, 47.1% of women from Europe/Central Asia reported experiences of spontaneous abortion, much higher than other regions. Those from Southeast Asia (28.5%) and Europe/Central Asia (22.8%) were also more likely to report ever having an abortion. In the sample, 2.1% of women reported female genital cutting, with 145 out of 162 women from Africa region. One in three women from Africa reported female genital cutting.

**Table 4:** Reproductive health indicators by Region of Birth (15+ years and older)

	Region of birth						
	Africa (Col %)	Southeast Asia (Col %)	Europe/Central Asia (Col %)	Latin America and the Caribbean (Col %)	South Asia (Col %)	Total (Col %)	*p*-value
Total	541 (100)	634 (100)	603 (100)	351 (100)	6622 (100)	8751 (100.0)	
Parity: number of births woman has had							0.000
0	235 (47)	196 (33.7)	91 (20.2)	150 (46)	2005 (34)	2677 (34.5)	
1	4 (0.8)	0 (0)	0 (0)	0 (0)	16 (0.3)	20 (0.3)	
2	108 (21.6)	123 (21.2)	134 (29.8)	68 (20.9)	1380 (23.4)	1813 (23.4)	
3+	153 (30.6)	262 (45.1)	225 (50)	108 (33.1)	2503 (42.4)	3251 (41.9)	
Mean (SD)	2.5 (3.3)	4.3 (4.3)	3.8 (3.4)	2.9 (3.8)	3.2 (3.5)	3.3 (3.5)	
Pregnancy result							0.000
Negative	385 (93.4)	505 (97.7)	349 (93.8)	262 (94.2)	4063 (92.3)	5564 (93.0)	
Currently Pregnant	27 (6.6)	12 (2.3)	23 (6.2)	16 (5.8)	340 (7.7)	418 (7.0)	
Experience of spontaneous abortion							0.000
No miscarriages	389 (82.4)	469 (87.8)	136 (52.9)	256 (82.3)	4087 (81.5)	5337 (81.0)	
Miscarriage	83 (17.6)	65 (12.2)	121 (47.1)	55 (17.7)	928 (18.5)	1252 (19.0)	
Abortion in lifetime							0.000
No	441 (96.7)	378 (71.5)	122 (77.2)	266 (87.5)	4436 (93.8)	5643 (91.4)	
Yes	15 (3.3)	151 (28.5)	36 (22.8)	38 (12.5)	291 (6.2)	531 (8.6)	
Female genital cutting							0.000
No	337 (69.9)	554 (99.5)	516 (99.6)	331 (99.4)	5926 (99.8)	7664 (97.9)	
Yes	145 (30.1)	3 (0.5)	2 (0.4)	2 (0.6)	10 (0.2)	162 (2.1)	
Hep B surface antigen							0.000
Non-reactive	523 (97.9)	595 (95.5)	581 (98.8)	344 (99.4)	6452 (99.2)	8495 (98.8)	
Reactive	11 (2.1)	28 (4.5)	7 (1.2)	2 (0.6)	54 (0.8)	102 (1.2)	
Hep C							0.000
Non-reactive	479 (96.8)	469 (98.5)	519 (97)	284 (99.6)	5430 (99)	7181 (98.7)	
Reactive	16 (3.2)	7 (1.5)	16 (3)	1 (0.4)	56 (1)	96 (1.3)	
HIV							0.000
Negative	517 (97.9)	622 (100)	590 (100)	340 (99.1)	6423 (100)	8492 (99.8)	
Positive	11 (2.1)	0 (0)	0 (0)	3 (0.9)	3 (0)	17 (0.2)	
Syphilis VDRL or RPR							0.693
Non-reactive	517 (99.2)	601 (99.7)	574 (99.7)	340 (99.7)	6327 (99.6)	8359 (99.6)	
Reactive	4 (0.8)	2 (0.3)	2 (0.3)	1 (0.3)	23 (0.4)	32 (0.4)	
Chlamydia^a^							0.000
Negative	136 (99.3)	127 (94.1)	121 (99.2)	103 (93.6)	1281 (99.5)	1768 (98.7)	
Positive	1 (0.7)	8 (5.9)	1 (0.8)	7 (6.4)	6 (0.5)	23 (1.3)	

Age ≥ 15 for all observations

^a^Age ≥ 16 to Age ≤ 25

In terms of screening tests, 1.2% of women were positive for Hepatitis B surface antigen and 1.3% was positive for Hepatitis C. Only 17 people tested positive for HIV (0.2%), 32 tested positive for syphilis (0.4%), and 23 tested positive for chlamydia (1.3%). Eleven out of the 17 people who tested positive for HIV came from Africa and 23 out of the 32 women who tested positive for syphilis came from South Asia.

Among girls younger than 15 years of age, 0.6% had reported female genital cutting, with 13 out of the 14 girls coming from Africa (Table 5). In terms of screening tests, 0.4% were reactive to Hepatitis B surface antigen and 0.6% reactive to Hepatitis C. Only two girls tested positive for HIV, one from Africa and one from South Asia. Only three girls reported syphilis within their first 90 days of arrival to the California.

**Table 5:** Screening tests by Region of Birth for Girls (< 15 years old)

	Region of Birth						
	Africa (Col %)	Southeast Asia (Col %)	Europe/Central Asia (Col %)	Latin America and the Caribbean (Col %)	South Asia (Col %)	Total (Col %)	*p*-value
Total	353 (100)	229 (100)	303 (100)	57 (100)	2577 (100)	3519 (100.0)	
Hep B surface antigen							0.175
Non-reactive	341 (98.8)	206 (100)	245 (99.6)	50 (100)	2151 (99.7)	2993 (99.6)	
Reactive	4 (1.2)	0 (0)	1 (0.4)	0 (0)	7 (0.3)	12 (0.4)	
Hep C							0.818
Non-reactive	285 (99.3)	87 (98.9)	136 (100)	36 (100)	1519 (99.4)	2063 (99.4)	
Reactive	2 (0.7)	1 (1.1)	0 (0)	0 (0)	9 (0.6)	12 (0.6)	
HIV							0.547
Negative	303 (99.7)	190 (100)	222 (100)	50 (100)	1883 (99.9)	2648 (99.9)	
Positive	1 (0.3)	0 (0)	0 (0)	0 (0)	1 (0.1)	2 (0.1)	
Female genital cutting							0.000
No	223 (94.5)	104 (100)	218 (100)	34 (100)	1713 (99.9)	2292 (99.4)	
Yes	13 (5.5)	0 (0)	0 (0)	0 (0)	1 (0.1)	14 (0.6)	
Syphilis VDRL or RPR							0.796
Non-reactive	92 (100)	105 (99.1)	62 (100)	29 (100)	545 (99.6)	833 (99.6)	
Reactive	0 (0)	1 (0.9)	0 (0)	0 (0)	2 (0.4)	3 (0.4)	

Age < 15 for all observations

### Health risk behaviors by region of birth

Among women aged 15 years and older, 8.5% had ever smoked (Table 6). The highest levels come from Latin America and the Caribbean with 11.1% of women indicating they had smoked. Less than 5% of women had ever drank alcohol. 5.8% of women had elevated glucose levels.

**Table 6:** Health risk behaviors by Region of Birth

	Region of Birth						
	Africa (Col %)	Southeast Asia (Col %)	Europe/Central Asia (Col %)	Latin America and the Caribbean (Col %)	South Asia (Col %)	Total (Col %)	*p*-value
Total^a^	541 (100)	634 (100)	603 (100)	351 (100)	6622 (100)	8751 (100.0)	0.000
Ever smoke^a^							
No	524 (97.6)	598 (95.4)	573 (95.7)	311 (88.9)	5943 (90.4)	7949 (91.5)	
Yes	13 (2.4)	29 (4.6)	26 (4.3)	39 (11.1)	630 (9.6)	737 (8.5)	
Current smoker^a^							
No	9 (69.2)	16 (55.2)	20 (76.9)	25 (64.1)	287 (45.6)	357 (48.4)	0.002
Yes	4 (30.8)	13 (44.8)	6 (23.1)	14 (35.9)	343 (54.4)	380 (51.6)	
One alcohol drink^a^							
No	526 (98)	583 (93)	573 (95.7)	310 (88.6)	6277 (95.5)	8269 (95.2)	0.000
Yes	11 (2)	44 (7)	26 (4.3)	40 (11.4)	296 (4.5)	417 (4.8)	
Elevated glucose							
Not elevated	608 (98.2)	604 (97.4)	780 (91.3)	311 (98.4)	6840 (93.7)	9143 (94.2)	0.000
Elevated	11 (1.8)	16 (2.6)	74 (8.7)	5 (1.6)	459 (6.3)	565 (5.8)	

^a^Age ≥ 15 years

## Discussion

This study provides timely data for health professionals and policy makers on the health profile of refugee women and girls entering California from 2013 to 2017. The data is novel and unique information to help tailor health and social services for refugee women. One of the most striking results is the high levels of trauma and persecution among refugee women: almost one-third of women reported experiences of trauma and over half reported persecution. Across all regions, 15% of women reported trauma due to loss of homes and sudden moves. Refugee women may experience this sudden life disruption for a number of reasons including civil wars, environmental disasters, and organized violence. Additionally, 3.5% reported harming or killing someone else and 9.5% reported trauma due to a death of a family member. These results are in line with other studies among refugees that find high levels of mental health concerns, with variability across regions of origin. One study found that approximately 23% of refugee women from multiple regions of the globe experienced emotional distress [18], while another study among Cambodian refugees found that all participants had suffered a traumatic event, with 99% experiencing near-death due to starvation and 90% reporting a family or friend murdered [19]. Region of origin is likely to play a significant role in the levels of and types of trauma experienced.

We also note important regional differences in regards to trauma. Women from Africa and Latin America and the Caribbean reported the highest levels of trauma, specifically due to weapon assault, physical assault, and sexual assault. The findings that women from Latin America and the Caribbean report the highest levels of violence (i.e. weapon, physical, and sexual assault) are in line with reports of a rise in unaccompanied minors to the US from parts of Latin America, specifically Honduras, El Salvador, and Guatemala [20]. The high number of unaccompanied minors raised international concern when children were forced to flee their homes due to violence and crime [21]. The three countries have witnessed a proliferation of gangs and drugs and report some of the highest homicide rates in the region. Reports suggest that children experienced death of family members, poverty, and recruitment into gangs, causing an influx of out-migration to the US [21].

Moreover, refugee women and girls from Latin America and the Caribbean and Africa also report high levels of sexual violence (17 and 10.5%, respectively). Past studies find that women and girls are more vulnerable to sexual violence particularly prior to and during the migration process [9, 11, 12]. Our data suggests that approximately 80% of refugee women and girls lived in a transit country before entering the US. We do not have data on when these traumatic experiences occur, but past studies suggest that living in a transit country before entering the US resulted in higher risk of preterm birth [10]. In transit countries, young women are targeted for sexual exploitation due to unstable livelihoods and lack of protection. Women may engage in transactional or survival sex, in exchange for food or protection [11].

Our study suggests that 7.0% of refugee women were pregnant within 90 days of arrivals. Resettlement agencies and health providers should be equipped with information about antenatal care, nutrition, and pregnancy to newly arrived women. The overall reported rate for having an abortion was 8.6% in our sample, much lower than the US where approximately 24% of women will have had an abortion by age 45 [22]. In this study, 19% of women reported ever having a miscarriage. It should be noted that there was a particularly high level of missing data for these two indicators. Because this is self-reported data, women may be seriously under-reporting these experiences due to feelings of stigma and shame, or not knowing they were pregnant.

The ability to disaggregate data by region also highlights important differences across regions, including female genital cutting. Experiences of female genital cutting are well documented in Africa, and our data suggests that one in three women from Africa report this practice. FGC is higher among those older than 15 years old at 2.1% compared to 0.6% of girls younger than 15 years old. Data from Africa suggests that there is a trend towards curbing the practice among younger cohorts compared to older cohorts; however, poorly educated women remain vulnerable to these practices [23]. Reports in the US suggests a rise in female genital cutting, doubling in the past decade, and have attributed these trends to a rise in immigration from African countries [24]. Although much lower compared to Africa, there were ten cases of female genital cutting from South Asia. This is in line with other research that has documented the rising concerns of female genital cutting in the region [25]. There is an urgent need for resettlement agencies and health professionals working with refugee women to be educated on the social and cultural causes of female genital cutting.

In regard to screening tests for infectious diseases for women aged 15 years and older, there were 102 cases of hepatitis B and 96 cases of hepatitis C. Hepatitis B was highest among Southeast Asian refugees (4.1%) as is corroborated by existing literature [26]. Additionally, there were only 17 cases of HIV, 32 cases of syphilis, and 23 cases of chlamydia. Practitioners working with refugee communities should be aware of knowledge and understanding of infectious diseases when counseling patients. This will impact adherence to treatment and compliance of long-term follow up care [27]. Long-term treatment is a complex process and building a trusting relationship where cultural values are respected is of utmost importance.

Overall, health risk behaviors were also quite low among refugee women and girls. For example, less than 9% had ever smoked, and 4.8% ever drank alcohol. In contrast, among women aged 35 years of age in the US, 24% had smoked cigarettes in the past 30 days and 13% of women reported heavy drinking in the past 30 days [28]. Moreover, 5.8% had elevated glucose levels, with the highest levels from Europe/Central Asia and South Asia (8.7 and 6.3%, respectively). These levels are lower compared to the US population, in which 9.4% of the population have diabetes [29], but higher compared to Iraqi refugees (aged 0–76+) who reported 2.7% with diabetes [30]. These individuals should be carefully monitored as elevated glucose levels may be a sign of diabetes and lead to cardiovascular diseases and other chronic conditions. Lack of physical activity, access to nutritional foods, and low acculturation are diabetes risk factors for refugees [31].

This study has a number of limitations. First, there may be serious under-reporting of self-reported data including experiences of abortions, miscarriages, female genital cutting, trauma and persecution. Questions around trauma and persecution may result in recall bias as they are asked to recall back to their childhood. In particular, women may not report traumatic events because it may be difficult for women to recall these memories, while other events such as sexual violence might be stigmatizing. Therefore, these are conservative estimates. Additionally, because visual checks were only performed in a few instances for female genital cutting, this study is not able to report on specific numbers of methods used. Additionally, while all clinics received training on the RHEIS system and questions, clinics may vary in terms of their reporting. There was also significant missing data, which is typical of routine monitoring data platforms. Moreover, while this paper provides data on female refugees, future analyses should link data of children to their parents to assess the role of family units on children’s health outcomes. As a subset of this research, unaccompanied minors are a particularly critical issue for public health practitioners.

## Conclusions

This study bridges existing gaps in the literature, including lack of population-based data on female refugee health, by providing an up-to-date general health status of refugee women and girls from many countries who arrived in California, the highest receiving state of refugees in the US. The data includes over 14,000 female refugees and is one of the first population-based datasets of refugee women and girls in the US; thus allowing comparisons across regions and health outcomes. Additionally, this study is quantitative in nature, producing a much larger dataset than previous qualitative studies on this population. To our knowledge, this is the first study to present California population-based data on experiences of trauma and persecution, reproductive health indicators, screening tests, and health risk behaviors by region of origin.

There are a number of programmatic recommendations for agencies and service providers working with refugee women and girls. These data support the growing need for trauma-informed care, or the need to recognize and respond to the widespread impact of trauma. Principles of a trauma-informed approach include safety, trustworthiness and transparency, peer support, collaboration and mutuality, empowerment voice and choice, and cultural, historical and gender identities [32]. This approach works with patients and their families in a way that is empowering and focuses on the relationship between trauma and symptoms of trauma such as depression, anxiety, and substance use. The effects of trauma may be particularly long-lasting for children [33], and interventions are needed specifically with child and adolescent populations.

Patients should also be informed of treatment guidelines and health information in a language that women can understand. In our sample, over 80% of refugee women needed interpreters at the time of their health assessment. Language barriers and inadequate communication in the healthcare setting may have significant consequences, including delayed care, lower use of preventive services, less adherence to treatment [34], lower prenatal care utilization and lower quality care [35].

This data highlights the need for ongoing routine data collection and health assessments, including longitudinal data that links to long-term outcomes. Particularly for indicators such as infectious diseases, local jurisdictions should have access to data in order to promptly diagnose and treat patients for potentially long-term illnesses. Understanding the unique health challenges of refugee women and girls will ultimately help agencies and health providers support this population in obtaining quality and appropriate care.

## Data Availability

The datasets generated and/or analysed during the current study are not publicly available due to additional ongoing analyses, but are available from the corresponding author on reasonable request.

## Abbreviations

CDC: Centers for Disease Control
CRHA: California Refugee Health Assessment
PRM: Population, Refugees, and Migration
RHAP: Refugee Health Assessment Program
RHEIS: Refugee Health Information System
SIVs: Special Immigrant Visas
UNHCR: United Nations High Commissioner for Refugees
VOT: Victims of Trafficking
